# The vomeronasal organ and incisive duct of harbor seals are modified to secrete acidic mucus into the nasal cavity

**DOI:** 10.1038/s41598-024-62711-x

**Published:** 2024-05-23

**Authors:** Daisuke Kondoh, Wataru Tonomori, Ryota Iwasaki, Jumpei Tomiyasu, Yuka Kaneoya, Yusuke K. Kawai, Shun Ikuta, Hayao Kobayashi, Mari Kobayashi

**Affiliations:** 1 Department of Veterinary Medicine, Obihiro University of Agriculture and Veterinary Medicine, Obihiro, Hokkaido Japan; 2 Department of Geology and Paleontology, National Museum of Nature and Science, Tsukuba, Ibaraki Japan; 3 Ashoro Museum of Paleontology, Ashoro, Hokkaido Japan; 4 Incorporated Non-Profit Organization, Marine Wildlife Center of Japan, Abashiri, Hokkaido Japan; 5 Faculty of Bioindustry, Tokyo University of Agriculture, Abashiri, Hokkaido Japan

**Keywords:** Brain, Olfaction, Pheromone receptor, Pinnipeds, Semiaquatic animals, True seals, Zoology, Animal physiology, Olfactory system, Olfactory bulb, Olfactory receptors

## Abstract

Most terrestrial mammals have a vomeronasal system to detect specific chemicals. The peripheral organ of this system is a vomeronasal organ (VNO) opening to the incisive duct, and its primary integrative center is an accessory olfactory bulb (AOB). The VNO in seals is thought to be degenerated like whales and manatees, unlike otariids, because of the absence of the AOB. However, olfaction plays pivotal roles in seals, and thus we conducted a detailed morphological evaluation of the vomeronasal system of three harbor seals (*Phoca vitulina*). The VNO lumen was not found, and the incisive duct did not open into the oral cavity but was recognized as a fossa on the anteroventral side of the nasal cavity. This fossa is rich in mucous glands that secrete acidic mucopolysaccharides, which might originate from the vomeronasal glands. The olfactory bulb consisted only of a main olfactory bulb that received projections from the olfactory mucosa, but an AOB region was not evident. These findings clarified that harbor seals do not have a VNO to detect some chemicals, but the corresponding region is a specialized secretory organ.

## Introduction

Most mammals receive chemical substances from the external environment via an olfactory system to recognize conspecifics, enemies, and prey. The olfactory system comprises the main olfactory and vomeronasal systems, and the vomeronasal system plays an important role in the detection of various species-specific chemicals^1^. The peripheral receptor in the vomeronasal system is the vomeronasal organ (VNO), which comprises a pair of tubular structures that are independent of the nasal cavity, and generally opens into the incisive duct in carnivores such as cats^2^, dogs^3^, foxes^4^, bears^5^, and ferrets^6^. The VNO has a lumen lined with sensory and nonsensory epithelia, has mucous vomeronasal glands in the submucosa, and is covered by the vomeronasal cartilage. The vomeronasal glands secrete mucous fluids into the lumen, and chemical substances such as pheromones are dissolved in the mucous fluid and then bind to vomeronasal receptor proteins expressed in the sensory epithelium.

The olfactory bulb (OB) is the primary center of the olfactory system in the brain. The large portion of the OB that receives projections from the olfactory mucosa within the nasal cavity is called the main olfactory bulb (MOB). Receptor neurons in the VNO project axons to the accessory olfactory bulb (AOB), which occupies a specific dorsocaudal region of the OB. The AOB is histologically independent of the MOB, and it structurally contacts the dorsal part of the lateral olfactory tract, which is the output pathway from the MOB^7,8^.

Most terrestrial mammals have a VNO^1,9–12^, but some marine mammals such as manatees^13^ and whales^14^, as well as some bats^15^ and catarrhine primates^16^, do not. Among these, the absence of morphological vomeronasal structures in marine mammals is widely explained by the understanding that chemical communication is not important for aquatic mammals^17^.

Pinnipeds are amphibious marine carnivores and are broadly divided into phocids and otariids. Because an AOB has been identified in some otariid pinnipeds (i.e., *Callorhinus ursinus*, *Eumetopias jubatus*, and *Zalophus californianus*)^7^, although no histological features were reported, otariids are thought to retain the functional VNO. On the other hand, the OB of phocids have so far been evaluated in only one harbor seal (*Phoca vitulina*), but no AOB have been found^7^. Recent findings of genes specific to the vomeronasal system have also suggested that the VNO has degenerated in harbor seals^18–20^. However, harbor seals have high olfactory sensitivity to atmospheric dimethyl sulfide, which is an indicator of their feeding zones^21^. Furthermore, nose-to-nose contact, especially between pups and their mothers^22^, implies interspecific chemical communication via the olfactory system in harbor seals. Here, we evaluated the detailed structure of the VNO and OB of harbor seals to consider the relationship between the olfactory system and the behavior in marine animals.

## Materials and methods

### Animals

Table 1 summarizes information about the three harbor seals used in this study. All individuals used in the present study died from causes unrelated to any research. Two seals were killed due to administrative population control in the national management plan, gross anatomical examination was performed, and then fresh tissue was fixed within 24 h postmortem for histological analysis. The remaining one died in the wild as bycatch, and the head was frozen and used for CT analysis. This study proceeded according to the Regulations on Management and Operation of Animal Experiments at Obihiro University of Agriculture and Veterinary Medicine (OUAVM), and the Animal Care and Use Committee of OUAVM approved the experimental protocol (notification no. 24-9).

**Table 1:** Computed tomographic and histological analyses of harbor seals.

ID	Sex	Status	Preservation	Computed tomography	Histology		Source
					Nasal cavity	Olfactory bulb	
A	Female	Subadult	Frozen	Yes	No	No	Bycatch
B	Female	Subadult	Fresh	No	No	Yes	Administrative control
C	Male	Adult	Fresh	No	Yes	Yes	Administrative control

### Computed tomography (CT) imaging

The frozen head was thawed, and CT images were acquired using an Aquilion TSX-201A scanner (Toshiba Medical Systems Corporation, Otawara, Japan) under the following conditions: 120 kV, 150 mA, and 0.5 mm-thick slices. Imaging data stored in DICOM format were processed using RadiAnt DICOM Viewer (https://www.radiantviewer.com). We evaluated serial frontal sections, as well as horizontal, sagittal, and frontal sections at the same point by using the “multiplanar reconstructions (3D MPR)” tool in the RadiAnt DICOM Viewer.

### Gross anatomy

The nose of a fresh specimen was dissected, and the regions around the incisive foramen were frontally sawed. The skulls of two specimens were sawed to expose the brain, and the most anterior parts of these brains including the OB were carefully removed.

### Histological analyses

The dissected nasal parts of a fresh specimen were fixed in 10% formalin for 1–2 weeks, decalcified in Plank-Rychlo solution (Fujifilm Wako Pure Chemical Corporation, Osaka, Japan) for 3 days, and then embedded in paraffin. The nasal tissues were frontally sliced into 5-μm-thick sections, deparaffinized, and stained with hematoxylin and eosin (HE), periodic acid-Schiff (PAS), or Alcian blue (AB; pH 1.0).

The OBs of the two fresh specimens were fixed in 10% formalin for 1 week and embedded in paraffin. The OBs were then sliced sagittally into 5-μm-thick serial sections at intervals of 50 μm, deparaffinized, and stained with HE.

### Analysis of the harbor seal genome assembly

We used TBLASTN with an e-value of < 1e-6 as the identity threshold for RNA and a pseudogene sequence database based on the harbor seal genome assembly (GCA_004348235.1) to identify vomeronasal receptor 1 (V1R) and 2 (V2R) genes, as previously described^23^. The genes were classified as intact genes including partial and full-length pseudogenes, according to the National Center for Biotechnology Information (NCBI) gene database.

## Results

### Location and histology of candidate sites for the VNO and incisive duct

**Figure 1:**
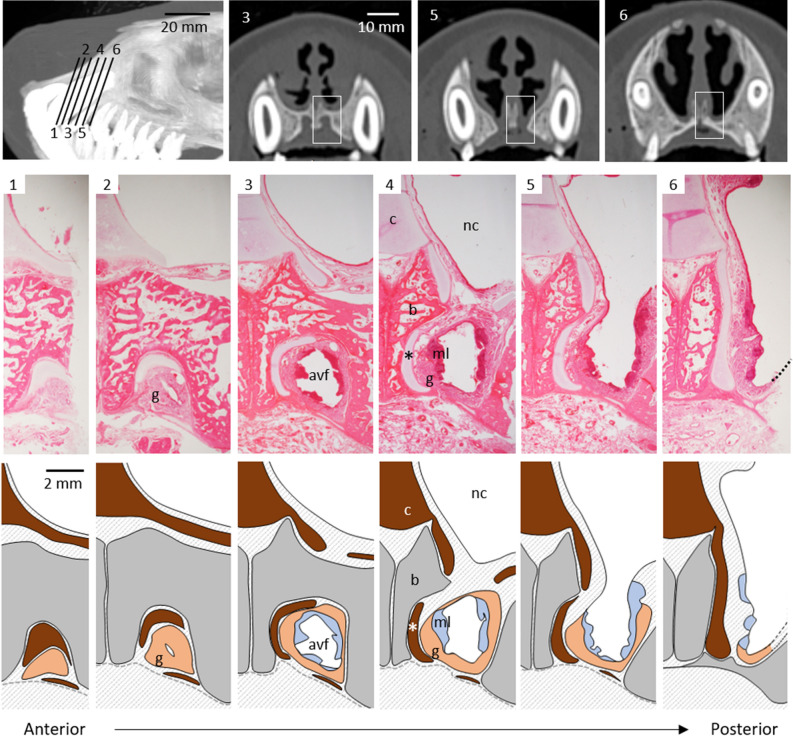
Location and structure of a candidate vomeronasal organ site in harbor seals. A pair of anteroventral fossae, in which incisive ducts expand and end at a blind end, are present between lines 1–6 on a computed tomographic (CT) lateral image (top left). CT cross sections 3, 5, and 6 (top right) correspond to lines 3, 5, and 6, respectively, and boxes indicate areas shown in histological images. An incisive foramen is observed in CT sections 5. Histological cross images 1–6 (HE staining) correspond to lines 1–6, with each schematic illustration below. *avf* anteroventral fossa, *b* bone, *c* septal cartilage, *g* mucous gland acini, *ml* mucosa-associated lymphoid tissues, *nc* nasal cavity; *vomeronasal cartilage.

The VNO of carnivores is located at the base of the nasal septum, and its anterior end opens into the incisive duct, as described above. In gross anatomy, although an incisive papilla was clear in the harbor seals, an opening of the incisive duct into the oral cavity was not found. Therefore, we indicated corresponding locations assuming the existence of an incisive duct (Fig. 1, lines 1‒6) according to the location of the incisive foramen by CT imaging to identify structures associated with the VNO (Supplementary Fig. S1).

**Figure 2:**
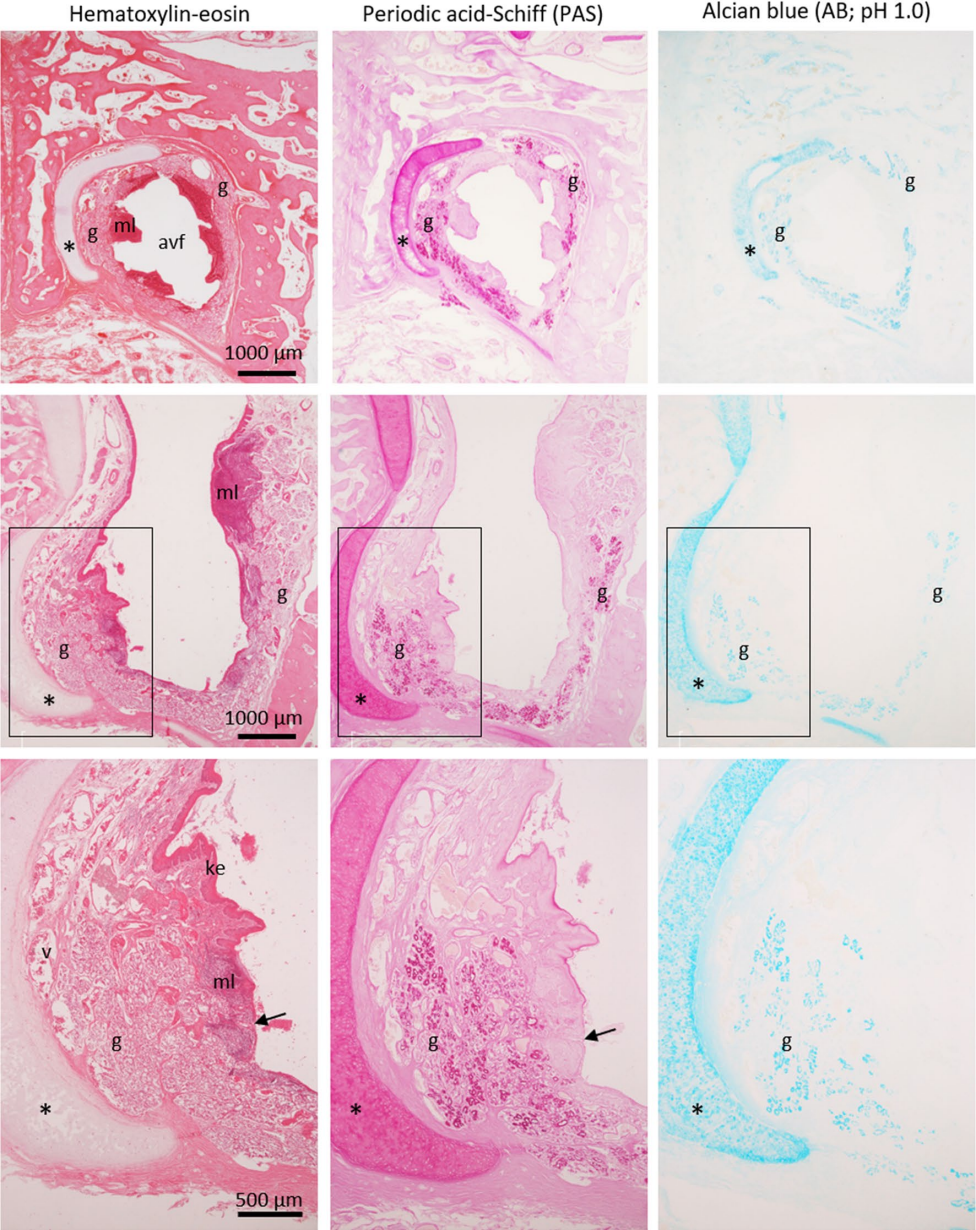
Histological features of an anteroventral fossa. Top and middle correspond to panels 3 and 5 in Fig. 1, respectively. Boxes indicate areas shown in lower panels. An anteroventral fossa (avf) is lined by keratinized epithelium (ke) and is parallel to a vomeronasal cartilage (asterisks). Some large veins (v), PAS- and AB-positive secretory gland acini (g), and mucosa-associated lymphoid tissues (ml) are found in submucosa. Arrows indicate an opening of a secretory duct.

Luminal structures lined by sensory or nonsensory epithelia of the VNO were absent, but degenerated vomeronasal cartilage was present medially (Figs. 1 and 2). The absence of a VNO lumen and the presence of vomeronasal cartilage were preliminary confirmed in two females (IDs: A and B) as well as a male used for histological analysis (ID: C). The area corresponding to the incisive duct communicated with the nasal cavity, extended in the anteroventral direction inside the incisive bone while maintaining a large space, and ended as a blind end (Fig. 1). We refer to this structure as the anteroventral fossa. The anteroventral fossa was lined by keratinized stratified epithelium and had mucosa-associated lymphoid tissues consisting of many lymphocytes (Figs. 1 and 2). This fossa was rich in PAS-positive mucous glands (Figs. 1 and 2), unlike the common nasal glands, which consisted of serous acini (Supplementary Fig. S2), and the secretory ducts opened into the nasal cavity (Fig. 2). We refer to these mucous glands as the anteroventral nasal glands. The anteroventral nasal glands were positive for AB (pH 1.0) (Fig. 2), indicating that these glands secrete acidic mucopolysaccharides containing sulfate into the nasal cavity. The submucosal tissue of this fossa contained many large blood vessels (supplementary Fig. S3), and no distinct nerve bundles were found (supplementary Fig. S4).

### Histology of the OB

The harbor seal OB comprised a small structure in the ethmoidal fossa at the anterior end of the brain, and the lateral olfactory tract extended ventrally from the OB (Supplementary Fig. S5). The dorsocaudal part of these structures was fixed to the cerebral hemispheres by the meninges, and cortical protuberances corresponding to the AOB were not evident (Supplementary Fig. S5).

**Figure 3:**
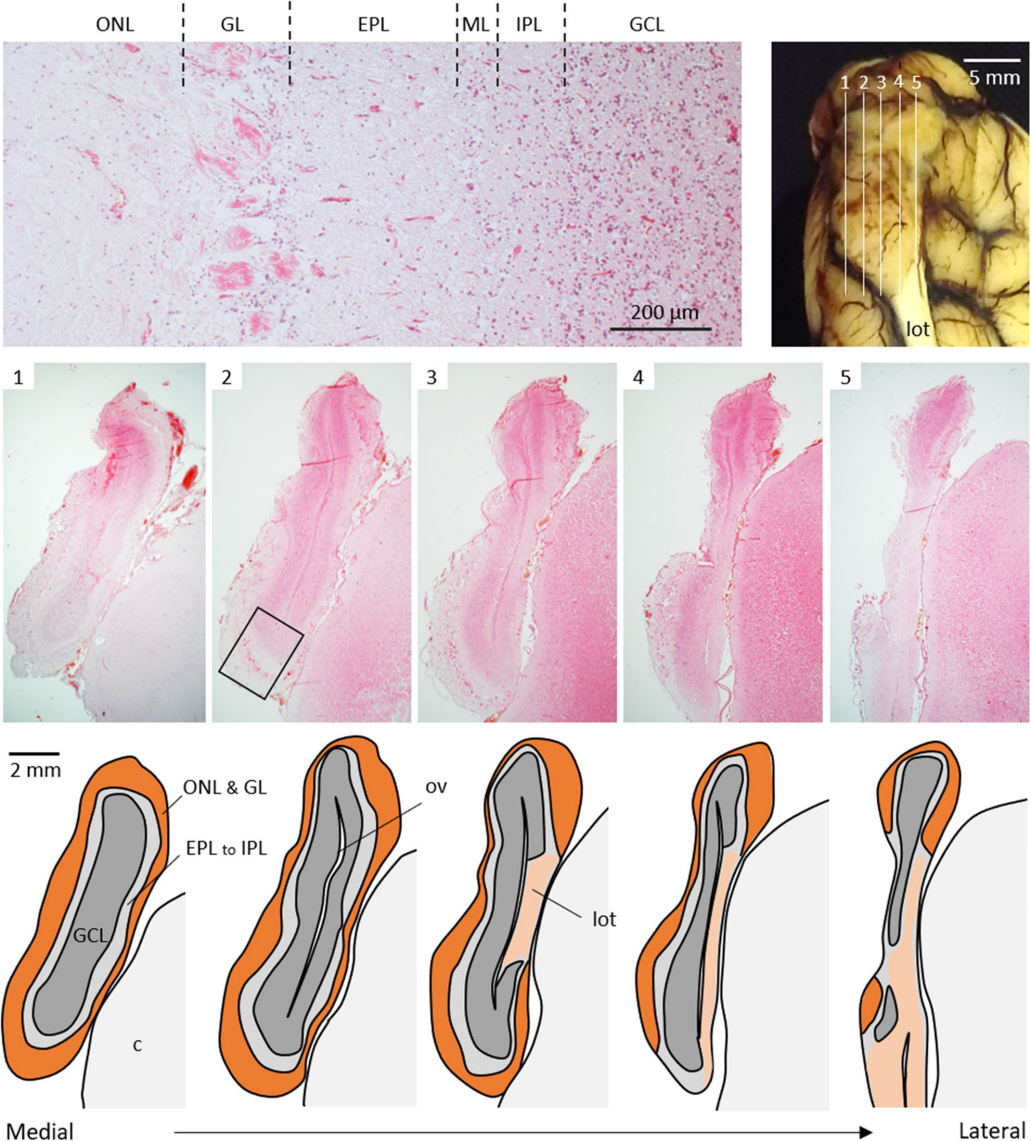
Histological structures of harbor seal olfactory bulb. An olfactory bulb comprises six layers (top left; HE staining), similar to a main olfactory bulb in most mammals. Lines 1–5 in frontal view of left olfactory bulb (top right) indicate areas corresponding to histological sagittal sections 1–5 (HE staining), respectively, with each schematic illustration below. A box indicates an area shown at top left. *c* cortex, *EPL* external plexiform layer, *GCL* granule cell layer, *GL* glomerular layer, *IPL* internal plexiform layer, *ML* mitral cell layer, *lot* lateral olfactory tract, *ONL* olfactory nerve layer, *ov*, olfactory ventricle.

The main part of the OB histologically consisted of the olfactory nerve, glomerular, external plexiform, mitral cell, internal plexiform, and granule cell layers (Fig. 3). This structural feature is consistent with that of general mammalian MOBs. The entire circumference of the medial half of the OB consisted of MOB layers (Fig. 3, Supplementary Figs. S6 and S7). Regions other than the posterodorsal region in the lateral half were also composed of MOB, but AOB structures were not found, even in the posterodorsal region occupied by the lateral olfactory tract (Fig. 3, Supplementary Figs. S6 and S7).

### Numbers of V1R and V2R genes

Our genome assembly analysis revealed that the harbor seal genome contained four intact (15 pseudogenes) V1R genes and no intact (a single pseudogene) V2R genes (Supplementary Table S1).

## Discussion

The vomeronasal system is an important chemosensory mechanism of intraspecific communication within various mammalian species. The vomeronasal system of only one individual harbor seal has been morphologically analyzed, and it was described as “*no AOF (Accessory Olfactory Formation)?*” in the OB^7^. On this basis, some publications have stated that true seals lack a VNO, and no morphological studies have been performed on the VNO. However, the presence or absence of a vomeronasal system in seals should be evaluated more carefully, because an AOB has been identified in some otariid pinnipeds^7^, indicating that a vomeronasal system is required for their ecology. The present findings showed in fine detail, that harbor seals do not have a VNO as a sensory organ in addition to an AOB.

Conspecific communication via pheromones in mammals seems to be related to the group formation. Among pinnipeds, three otariids (i.e., *C. ursinus*, *E. jubatus*, and *Z. californianus*) are estimated to receive pheromones through the vomeronasal system^7^, and they form harems. On the other hand, harbor seals do not form harems. In order to clarify whether the presence or absence of the vomeronasal system reflects differences in lineage or harem formation, it is required to examine elephant seals (*Mirounga angustirostris* and *M. leonina*) that form harems.

The absence of a vomeronasal system for receiving chemical information means that no pheromonal responses trigger specific behaviors or physiological states in harbor seals. However, these findings do not rule out the possibility that the main olfactory system is used for conspecific recognition. We detected four predicted V1R genes in the genome, which may be capable of receiving pheromone-like stimuli. In goats, some V1Rs are expressed not only in the VNO, but also in the olfactory mucosa lining the nasal cavity^24^. Despite the small OB of harbor seals, the structurally layered MOB was histologically similar to that of carnivores such as dogs. The main olfactory system, rather than the vomeronasal system, might function in conspecific recognition, such as mother and pup communication via nose-to-nose contact^19^.

**Figure 4:**
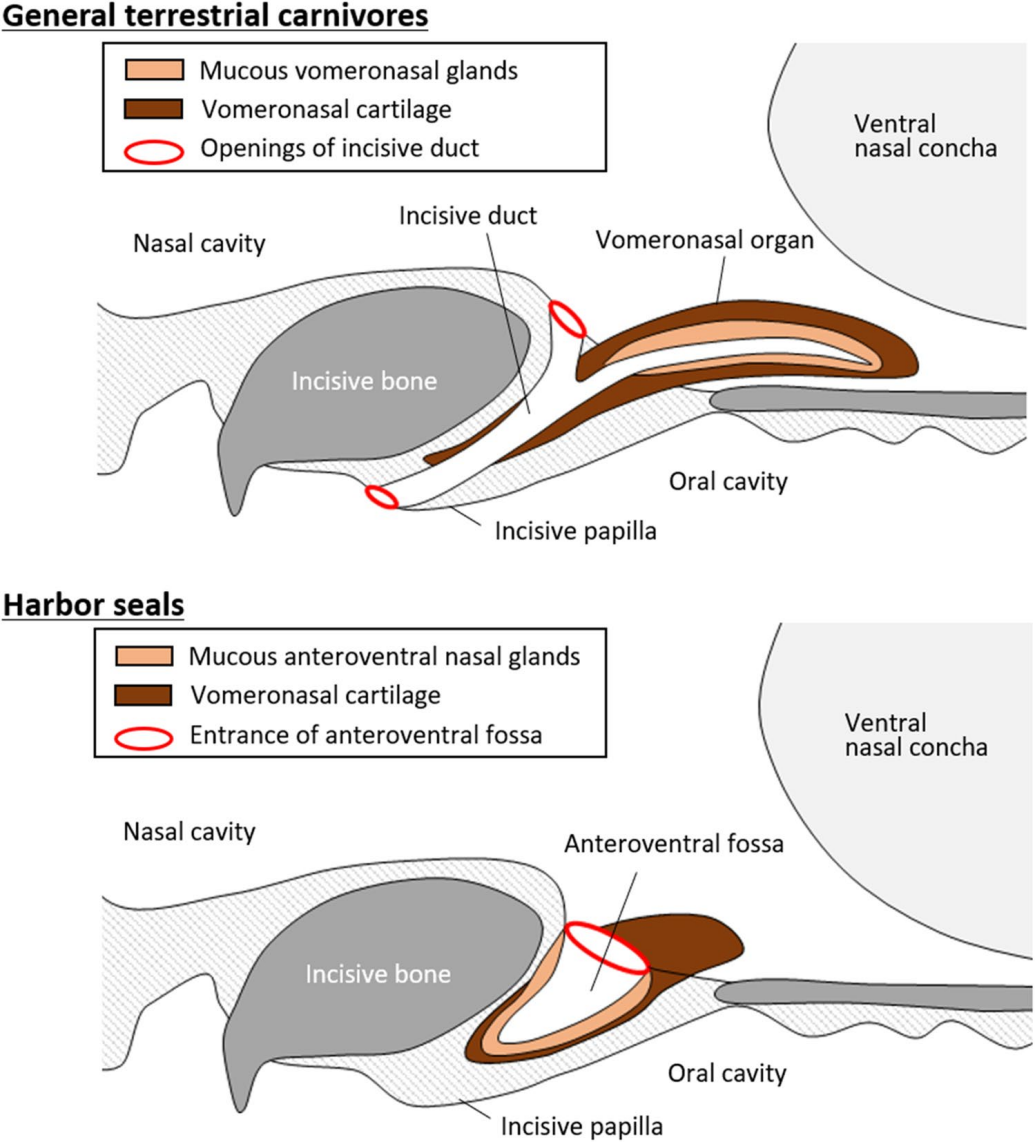
Comparison of structures related to a VNO in terrestrial carnivores and harbor seals.

On the other hand, an interesting structure was observed at the candidate site of the harbor seal VNO. The dilated incisive duct communicating with the nasal cavity ended at a blind end, which was recognized as the anteroventral fossa (Fig. 4). This anteroventral fossa is a specialized secretory organ containing many mucous gland acini, unlike the common nasal glands that are mostly composed of serous glands. Because the vomeronasal glands of carnivores are mucous^25^, mucous acini of the anteroventral nasal gland may originate from the vomeronasal glands. The degenerated vomeronasal cartilage that runs parallel to the fossa also suggested that this secretory organ may be related to the VNO (Fig. 4).

The anteroventral nasal gland acini were positive for AB (pH 1.0), indicating that these glands secret mucopolysaccharides containing sulfate into the nasal cavity. We recently identified a specialized secretory structure with sulfate-containing acidic mucopolysaccharides in the nasal vestibular region of sea turtles^26^. Since charged substances are difficult to dissolve in acidic mucous fluids^27^, these secretions near the nostril may be suitable for protecting the nasal mucosa from salt in seawater. In other words, it is possible that the vomeronasal complex in harbor seals is modified from a sensory organ to a secretory organ to adapt to marine life. To confirm this, we need to track changes in the developmental stages of harbor seals. In addition, several mammals with a VNO, like horses^28^, also have the closed incisor duct, and thus comparison of their formation and function between seals and horses may help understand the variation of the VNO and the incisor duct in mammals.

Chemical communication via the vomeronasal system is widely considered to be insignificant for aquatic mammals^17^. The findings obtained for harbor seals in the present study support this notion. However, some otariids^7^, hippopotami^29^, and beavers^23^ have an AOB, and this supports the notion that pheromone communication occurs via the vomeronasal system in these amphibious animal species. Furthermore, the present findings suggested that the VNO of mammalian species without AOB has not simply disappeared, but may be modified as a secretory organ (Fig. 4). The vomeronasal system of individual amphibious animal species should be carefully evaluated.

### Supplementary Information

Supplementary Information.

## Data Availability

Data that support the study findings are available from the corresponding author upon reasonable request.
