# Supplementary material for: The vomeronasal organ and incisive duct of harbor seals are modified to secrete acidic mucus into the nasal cavity
Source: Sci Rep. 2024 May 23;14:11779. doi: 10.1038/s41598-024-62711-x (PMC11116377; doi:10.1038/s41598-024-62711-x)
Supplement: Supplementary file 1 — Supplementary Information. [file 41598_2024_62711_MOESM1_ESM.pdf]

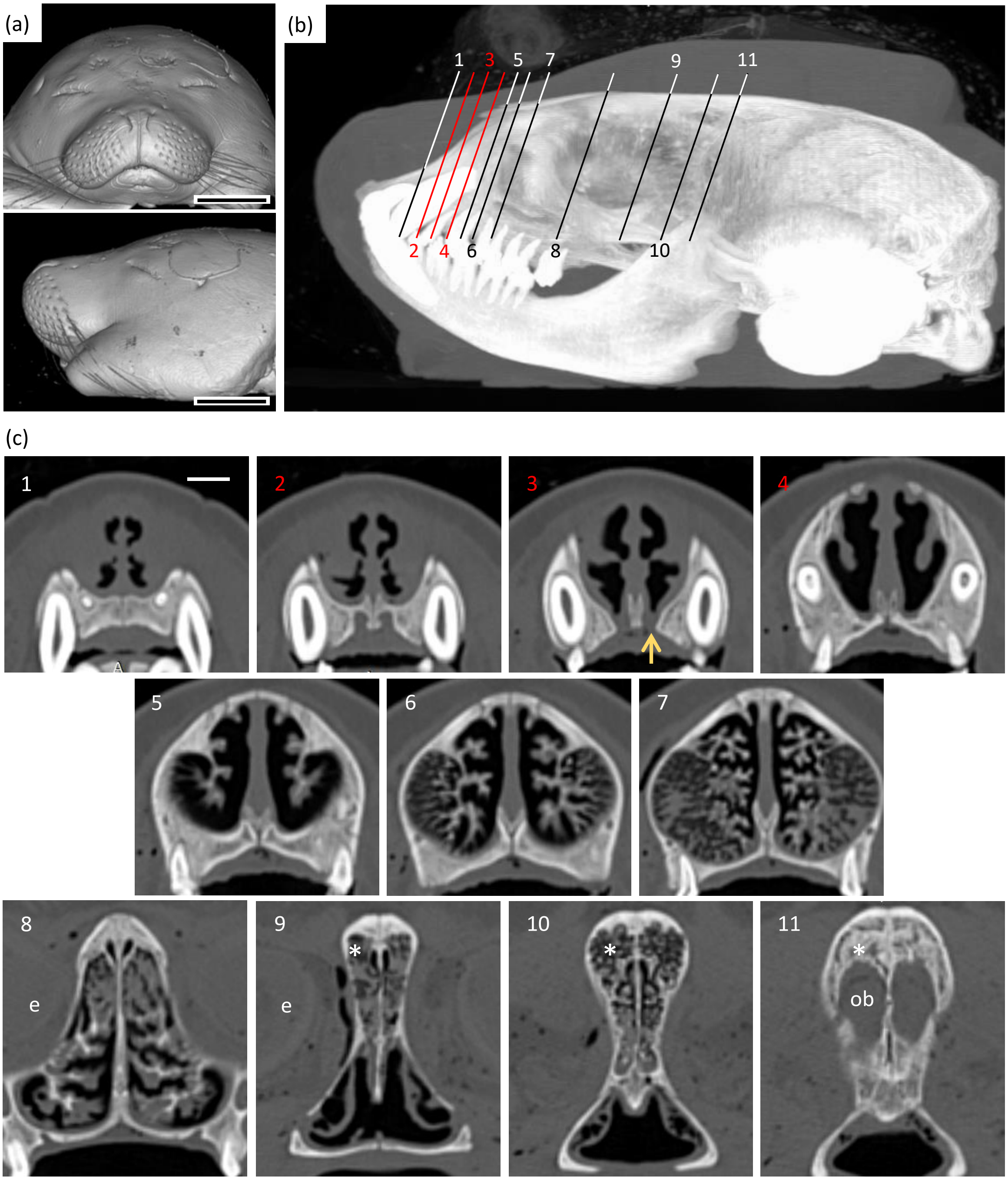

Supplementary Figure S1. Computed tomographic images of harbor seal nasal cavity. (**a**) Frontal (upper) and left lateral (lower) views of skin surface of head. (**b**) Left lateral image of internal skull structure. Lines 1–11 correspond to panels 1–11 in (**c**). Area between lines 2 and 4 is candidate vomeronasal organ (VNO) site. (**c**) Frontal views at various levels in anteroposterior axis. Arrow in panel 3 indicates incisive foramen. *Ethmoid turbinate; e, eye ball; ob, olfactory bulb. Bars = 50 (**a**) and 10 (**c**) mm.

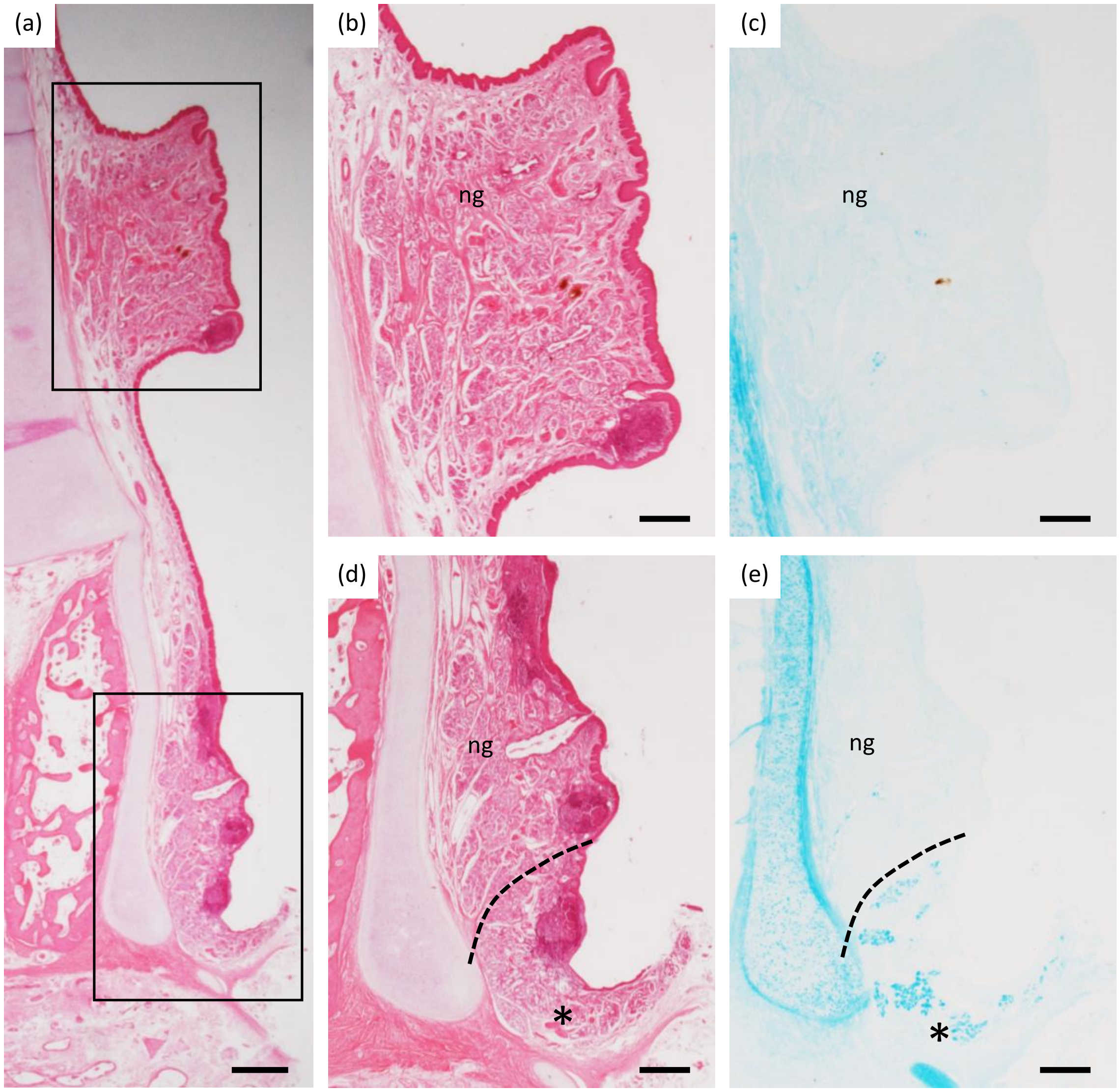

Supplementary Figure S2. Comparison of acini between anteroventral nasal glands and common nasal glands.

(**a**) HE stained section corresponding to panel 6 in Figure 1. (**b** and **d**) High magnified images of upper and lower boxes in (**a**), respectively. ng, serous common nasal glands; *mucous anteroventral nasal glands. (**c** and **e**) Alcian blue (pH 1.0) stained images corresponding to (b and d), respectively. Bars = 1000 (**a**) and 500 (**b**–**e**) mm.

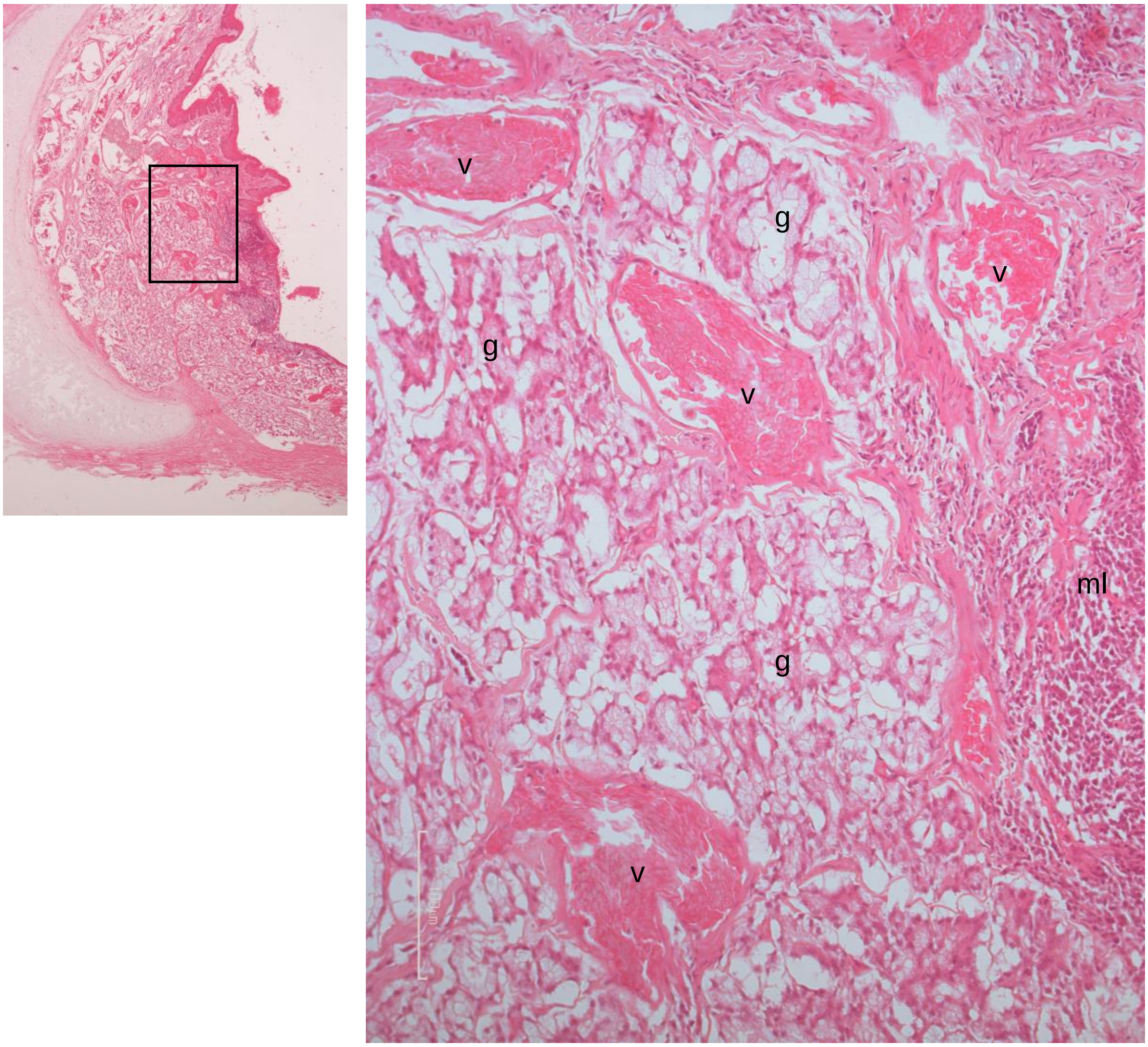

Supplementary Figure S3. Histological features of submucosal tissue in anteroventral fossae. High magnified image (right panel) indicated by a box in left panel. g, glands; ml, mucosa-associated lymphoid tissues; v, blood vessel.

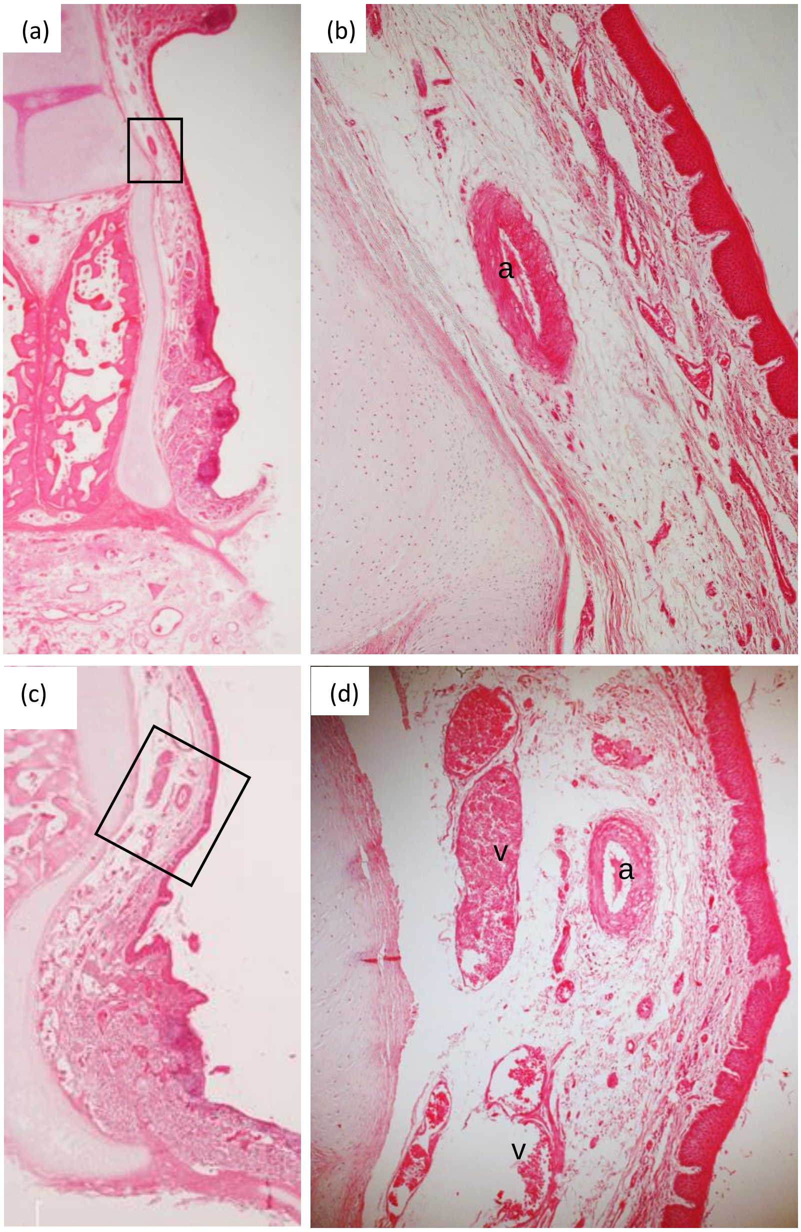

Supplementary Figure S4. Blood vessels containing blood cells that look like nerve bundles. (**a** and **b**) Region corresponding to panel 6 in Figure 1. (**c** and **d**) Region corresponding to middle panels in Figure 2. Boxes in (**a** and **c**) indicate the areas shown in (**b** and **d**), respectively.
a, artery; v, vein.

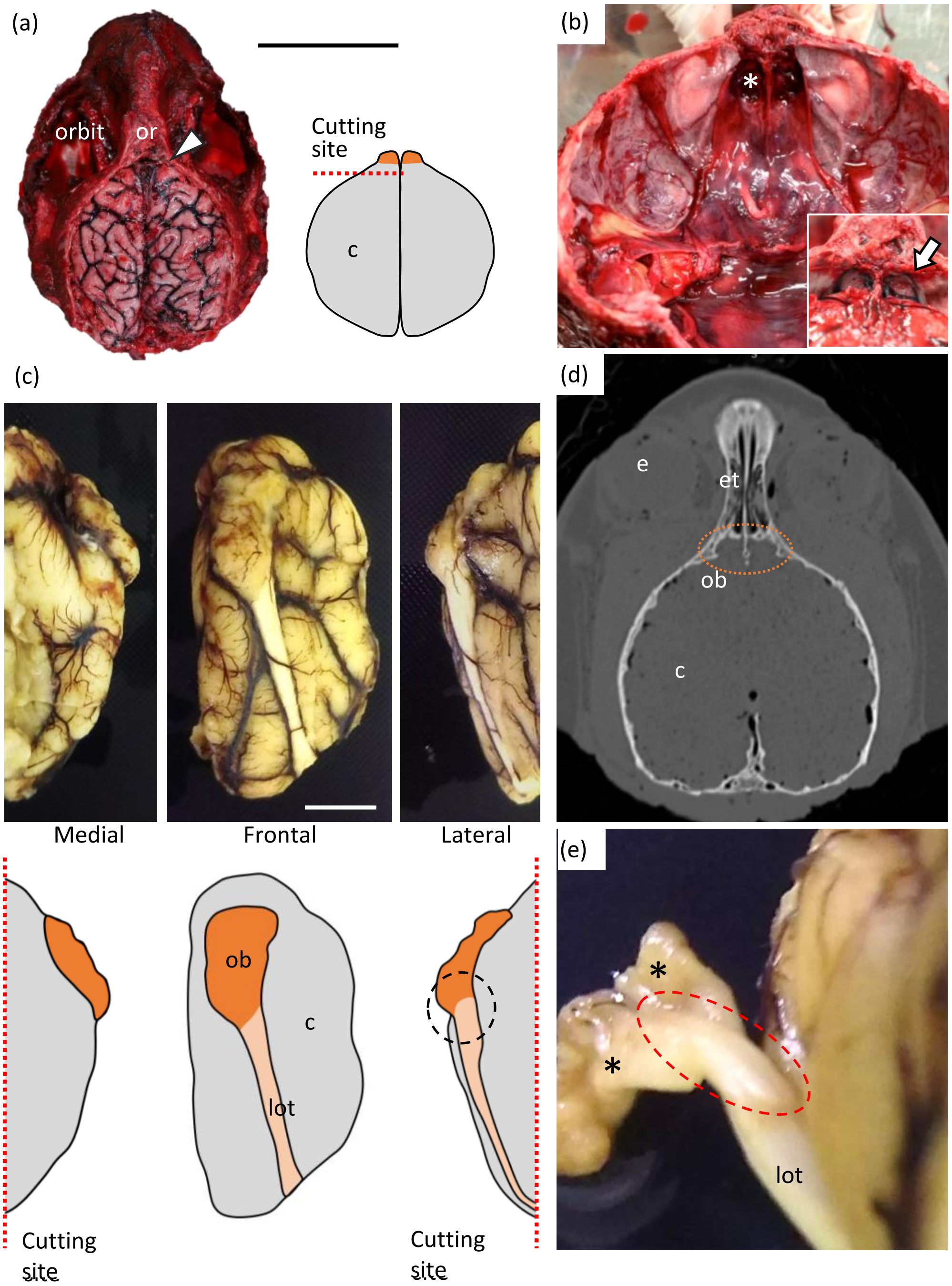

Supplementary Figure S5. Morphological structure of olfactory bulb (OB) in harbor seals. (**a**) Dorsal view of head after bone removal (left) and schematic illustration of OB location and size (right). Arrowhead in left and orange region in right indicate OB location. Red dashed line indicates cutting site for histological analysis of OB. c, cerebrum hemisphere; ro, olfactory region of nasal cavity. (**b**) Internal view of skull after brain removal. Insert, OB (arrow) in *ethmoidal fossae. (**c**) Medial, frontal, and lateral views (upper) and schematic illustrations (lower) of OB and lateral olfactory tract (lot). Black dashed circle corresponds to region in (**e**). Lines 1–5 correspond to panels 1–5 in Figure 4b. (**d**) CT image of horizontal section at OB level. e, eyeball; et, ethmoidal turbinate. (**e**) Dorsal view of lateral olfactory tract (lot) after removing meninges and inverting OB rostrally. Red dashed circle, candidate accessory olfactory bulb (AOB) region, but cortical protuberance is not evident. Bars = 50 (**a**) and 10 (**c**) mm.

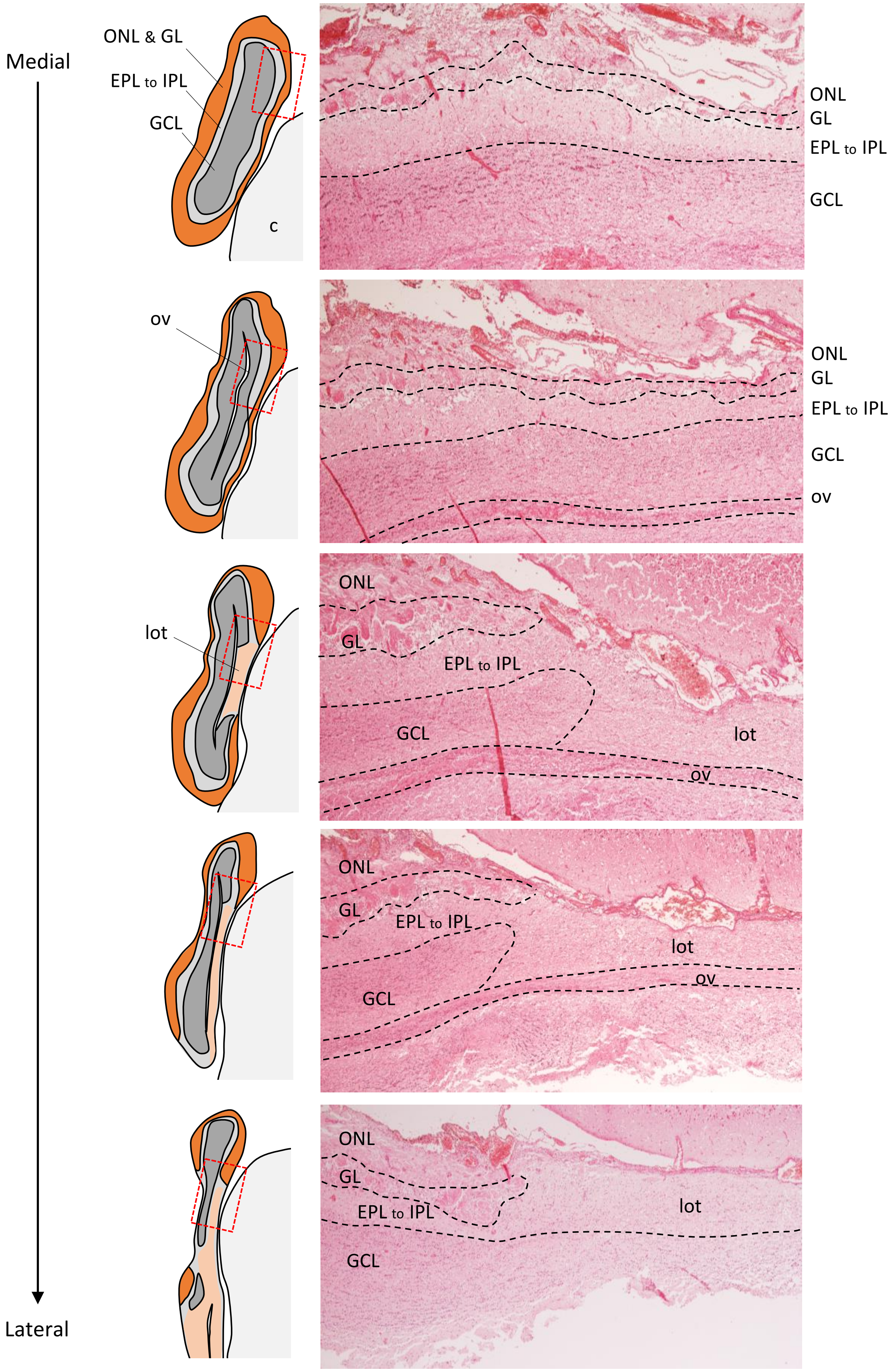

Supplementary Figure S6. High magnified images of posterodorsal part of OB.
Each image corresponds to a dashed box in the schematic diagram on the left. At all stages of a mediolateral axis, no AOB structure was found. Abbreviations are same as Figure 3.

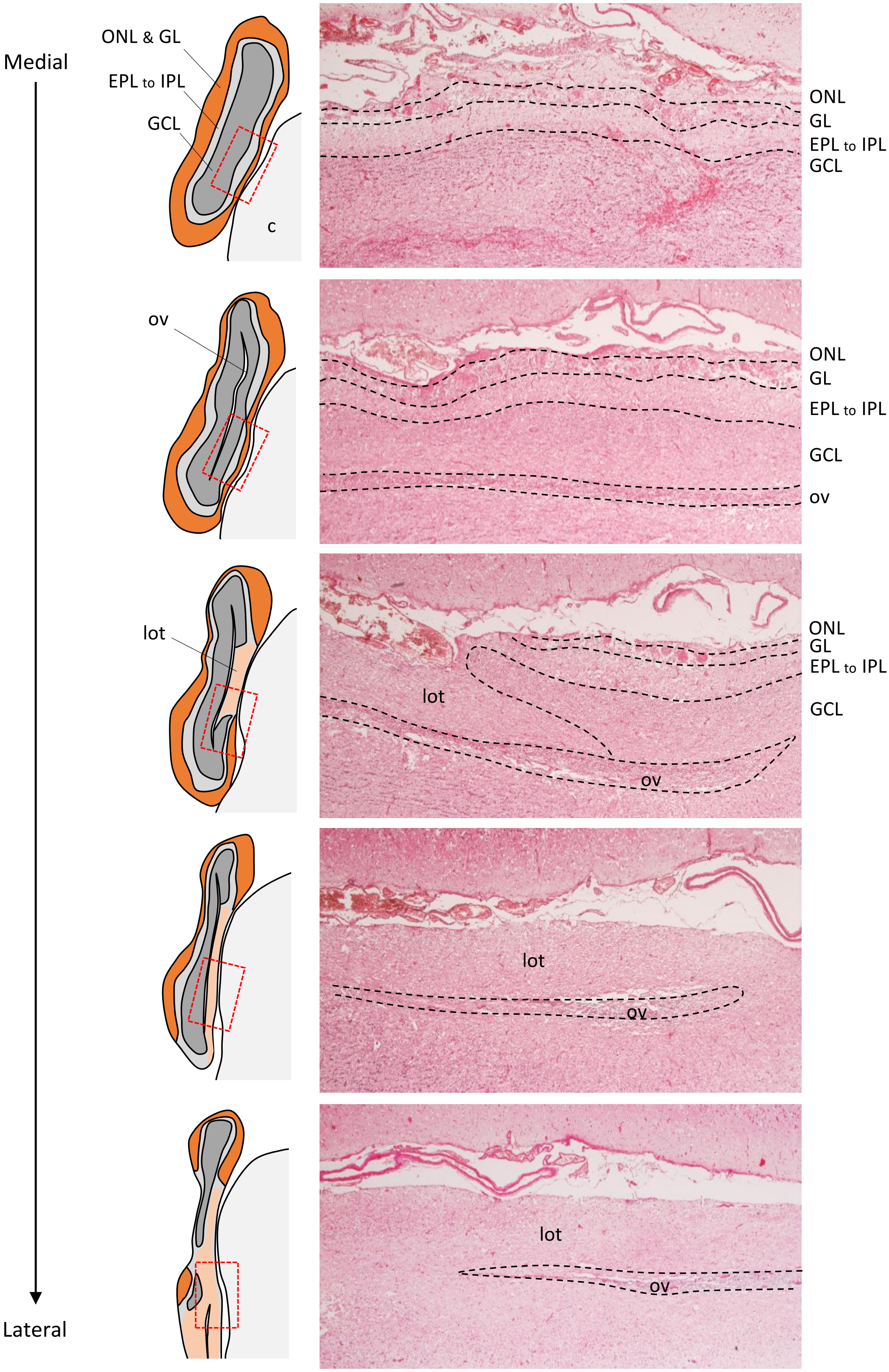

Supplementary Figure S7. High magnified images of posteroventral part of OB.
Each image corresponds to a dashed box in the schematic diagram on the left. At all stages of a mediolateral axis, no AOB structure was found. Abbreviations are same as Figure 3.

Supplementary Table S1. V1R and V2R genes encoded in the genome of harbor seals.

Gene ID	Type	GenBank accession	State
116622362	V1R	XM_032388820.1	Protein coding
116622453	V1R	XM_032388925.1	Protein coding
116622454	V1R	XM_032388926.1	Protein coding
116628657	V1R	XM_032399021.1	Protein coding
116622045	V1R		Pseudo
116622114	V1R		Pseudo
116622208	V1R		Pseudo
116622210	V1R		Pseudo
116622211	V1R		Pseudo
116622213	V1R		Pseudo
116622289	V1R		Pseudo
116622414	V1R		Pseudo
116622417	V1R		Pseudo
116622451	V1R		Pseudo
116623193	V1R		Pseudo
116625887	V1R		Pseudo
116628455	V1R		Pseudo
116629485	V1R		Pseudo
116629587	V1R		Pseudo
116634879	V2R		Pseudo
